# Promoting health equity: WHO health inequality monitoring at global and national levels

**DOI:** 10.3402/gha.v8.29034

**Published:** 2015-09-18

**Authors:** Ahmad Reza Hosseinpoor, Nicole Bergen, Anne Schlotheuber

**Affiliations:** Department of Health Statistics and Information Systems & Gender, Equity and Human Rights Team, World Health Organization, Geneva, Switzerland

**Keywords:** dimensions of inequality, heath equity, health inequality, monitoring, sustainable development goals, World Health Organization

## Abstract

**Background:**

Health equity is a priority in the post-2015 sustainable development agenda and other major health initiatives. The World Health Organization (WHO) has a history of promoting actions to achieve equity in health, including efforts to encourage the practice of health inequality monitoring. Health inequality monitoring systems use disaggregated data to identify disadvantaged subgroups within populations and inform equity-oriented health policies, programs, and practices.

**Objective:**

This paper provides an overview of a number of recent and current WHO initiatives related to health inequality monitoring at the global and/or national level.

**Design:**

We outline the scope, content, and intended uses/application of the following: *Health Equity Monitor* database and theme page; *State of inequality: reproductive, maternal, newborn, and child health* report; *Handbook on health inequality monitoring: with a focus on low- and middle-income countries*; *Health inequality monitoring* eLearning module; *Monitoring health inequality: an essential step for achieving health equity* advocacy booklet and accompanying video series; and capacity building workshops conducted in WHO Member States and Regions.

**Conclusions:**

The paper concludes by considering how the work of the WHO can be expanded upon to promote the establishment of sustainable and robust inequality monitoring systems across a variety of health topics among Member States and at the global level.

Health inequities are unjust and avoidable differences in health that stem from some form of discrimination or lack of access to certain resources. Often, inequitable health outcomes are systematically experienced by population subgroups that are disadvantaged as a result of factors, such as economic status, education level, sex, place of residence, race, ethnicity, age, or disability status. Advocates of health equity strive to improve the situation of disadvantaged subgroups by ensuring that all have access to necessary health interventions, and addressing the underlying causes of discrimination and disadvantage. Taking meaningful action toward achieving equity in health is a moral imperative, but it may also impart other benefits to society through enhancing population health, improving economic and living conditions, and advancing social justice.

From its inception in 1948, the World Health Organization (WHO) has endorsed health as a fundamental right for all, regardless of race, religion, political belief, economic status, or social condition (1). Henceforth, the WHO has repeatedly reaffirmed this commitment to improving the health of disadvantaged populations. For instance, the WHO promoted the importance of the *Declaration of Alma-Ata*. Adopted in 1978, the *Declaration of Alma-Ata* advocates for action to promote the health of all people of the world, acknowledging the need to reduce inequalities within and between countries (2, 3). The *Global strategy for health for all* was adopted by the World Health Assembly in 1981, prioritizing the achievement of equity in the way that health resources and health care are distributed and accessed (4). In more recent years, the Commission on Social Determinants of Health was established by the WHO to explore the root causes of inequities in health, and spur a global movement to achieve health equity (5).

Now, in light of the emerging post-2015 sustainable development goals (SDGs), the importance of equity is gaining attention as a cross-cutting theme for all development-related spheres, including health (6). The proposed SDG 10 calls for action to ‘reduce inequality within and among countries’. Indeed, this aim can – and should – be readily applied to all health-related aspects of the SDGs, but it is integrally linked to SDG target 3.8, which promotes the achievement of universal health coverage (UHC). The fundamental components of UHC are to ensure that all people who need health services are able to get them, and to enable access to these services without undue financial hardship (7, 8). An equity-oriented approach to the progressive realization of UHC calls for accelerated and early gains among disadvantaged subgroups, thereby improving the overall situation alongside reductions in inequality (9). Recommendations to measure progress toward UHC are based on monitoring health inequalities (10).

## Health inequality monitoring

Measuring health inequalities – the observed differences in health between subgroups – is a metric through which the normative concept of health equity can be evaluated (11). Measuring inequality between subgroups requires the use of health data that are disaggregated according to a relevant dimension of inequality (i.e. demographic, socio-economic, or geographical factor). Disaggregated data break down national averages, helping to expose patterns of how indicators of health are distributed throughout a population. Understanding these patterns serves to inform how policies, programs, and practices can be aligned to promote better health among the disadvantaged. The practice of data disaggregation is acknowledged as a key principle for sustainable development (6, 12).

Increasingly, the field of knowledge surrounding measuring and monitoring health inequality, as well as the resources dedicated to the practice, is expanding; a number of ‘best practice’ recommendations and overviews have been published (13–17). Comprehensive discussions about the technical complexities of health inequality monitoring are outside of the scope of this paper; however, we acknowledge that this is a deservedly growing area of research and debate.

Health information systems refer to the collection, analysis, and reporting of health data – major components which underlie health inequality monitoring (13). Equity-oriented health information systems have data collection practices that facilitate data disaggregation by relevant dimensions of inequality and across a wide selection of health topics; have the knowledge, expertise, and resources to conduct and interpret standardized analyses of health inequalities; and produce regular and high-quality reports of the state of inequality. The framework below classifies resources related to health inequality monitoring according to their key characteristics (Table 1).

**Table 1:** Framework to classify resources related to health inequality monitoring

Level of monitoring	Component of monitoring		

	Collection	Analysis	Reporting
Global level	Data collection practices are standardized across countries, and data are comparable.	Standardized measures are comparable across borders and over time.	Reports present the global situation of inequality. Comparisons across countries enable benchmarking, and allow countries to learn from one another. Global reports may have implications for broader level resource allocation and are important for tracking progress on global targets.
	Common data sources for global monitoring include household surveys. Data about dimensions of inequality should be relevant across all study countries (e.g. household wealth quintiles, place of residence, education level and sex). Common definitions for health indicators should be adopted.	Analyses calculate cross-country comparisons of within-country inequalities.	
		Data are disaggregated according to a common set of dimensions of inequality.	
National level	Data collection covers health indicators and dimensions of inequality that are relevant within the country.	Analyses calculate within-country inequality.	Reports present the national situation of inequality and can account for unique contextual factors.
	Data sources may include censuses, vital registrations, facility records, and national surveys.	Data are disaggregated according to dimensions of inequality that are most applicable to the country.	National reports may have direct implications for health information system strengthening and equity orientation of national policies, programs, and practices.

Recognizing the importance of health inequality monitoring to inform equity-oriented health policies, programs, and practices, the WHO has developed a number of resources to build capacity for global and national health inequality monitoring. This paper provides an overview of WHO health inequality monitoring products related to global and/or national monitoring, and addressing data collection, analysis and/or reporting. It includes a brief description of the scope, contents, and intended uses/application of each product. Then, we consider the upcoming work of the WHO in the area of health inequality monitoring, and how the organization can support sustainable progress toward developing effective health inequality monitoring systems.

## Global health inequality monitoring

Global health inequality monitoring can be described as a comparison of within-country inequality across countries, and it is a useful practice to track progress on international initiatives such as the SDGs. The results of global health inequality monitoring may inform high-level decisions about resource allocation, and identify areas in need of additional support. Global-level monitoring enables benchmarking among countries, prompting poorly performing countries to recognize areas for improvement, and draw lessons from the success stories of better-performing countries.

In order to make valid global comparisons of health inequality monitoring across settings, data must be comparable. Dimensions of inequality should be selected based on their appropriateness, relevancy, and data availability across all countries, and may include economic status, education level, place of residence, and sex (13).

In support and promotion of global health inequality monitoring, WHO products aim to provide disaggregated data that are comparable across countries, exemplify how global monitoring can be conducted and reported, and/or encourage discussion about the context and conduct of global health inequality monitoring.

## National health inequality monitoring

Health inequality monitoring at a national level helps to evaluate the impact of policies, programs, and practices on disadvantaged subgroups. This priority is reflected in the proposed SDG target 17.18, which calls for countries to increase the availability of data disaggregated by income, sex, age, race, ethnicity, migratory status, disability, geographic location, and other characteristics relevant in national contexts (6). National health inequality monitoring is the basis for global monitoring, but it also goes beyond this to seek a more sophisticated understanding of the health status of disadvantaged subgroups within a country. National health inequality monitoring can be tailored to investigate health indicators and dimensions of inequality that may be relevant within a particular country, but not universally. The process can make use of the best available data sources at a national level, such as certain administrative data, censuses, vital registrations, and national surveys; these sources may not be appropriate to generate comparable figures across countries (i.e. for global monitoring).

Products and activities aimed at a national-level advocate for a sustainable and systematic approach to national inequality monitoring, and/or build technical knowledge for the process of health inequality monitoring. The dissemination of these products and delivery of these activities strive to reach many countries across all WHO Regions.

## WHO global and national health inequality monitoring products

### Health Equity Monitor database and theme page

Global and national level: analysis and reporting

*Health Equity Monitor* theme page: www.who.int/gho/health_equity/en/

*Health Equity Monitor* data repository: apps.who.int/gho/data/node.main.HE-1540?lang=en

**Scope**: The *Health Equity Monitor* database and theme page were launched in 2013, as one component theme within the WHO *Global Health Observatory*. The aim of the *Health Equity Monitor* is to serve as a platform to enable global or national health inequality monitoring. Comparable data on the topic of reproductive, maternal, newborn, and child health (RMNCH) are available across a large number of countries; RMNCH is currently the featured topic of the *Health Equity Monitor*.

Regular updates to the *Health Equity Monitor* are done approximately once per year to expand the database with newly available data and update the associated texts and visuals. Updates are done in coordination with the International Center for Equity in Health at the University of Pelotas in Brazil, where the data analyses are performed.

**Contents**: The *Health Equity Monitor* data repository contains data for more than 30 RMNCH indicators, disaggregated by education, economic status, place of residence (rural and urban), and child's sex (where applicable). Data were taken from nearly 250 surveys done in 94 countries over 1993–2013. For almost three-fourths of countries, disaggregated data are available for at least two time points. Comprehensive information about the data, including detailed indicator definitions and links to survey methodology, can be accessed directly from the data repository tables.

The various contents of the *Health Equity Monitor* theme page highlight messages from the database, and support the interpretation and reporting of health inequality monitoring. Interactive visuals communicate inequalities in selected health interventions and outcomes, demonstrating the latest situation within and across countries, and changes in inequality over time. Each interactive visual is accompanied by a feature story that provides a comprehensive assessment of a selected health indicator and dimension of inequality combination.

Equity country profiles are available for 86 countries, which allow disaggregated data to be compared across indicators and over time for a given country. Country profiles can be customized by dimension of inequality, RMNCH indicator category, and survey.

A compendium of indicator definitions is available, detailing the calculation of each indicator in the database. Additionally, a range of WHO products related to health inequality monitoring are made available through the theme page.

**Intended uses**: The contents of the *Health Equity Monitor* appeal to a range of potential audiences. The data repository is particularly useful for technical audiences that may wish to download data tables for further analyses of inequality at global or national levels. The theme page supports both technical and non-technical audiences, providing entry points into the database through interactive visuals and feature stories. Benchmarking can be done for countries of interest, and comparisons may be made based on dimensions of inequality, health indicators, WHO Regions, and country income groups. In a broad sense, the elements of the theme page demonstrate best practices in reporting health inequality, and may be useful examples for audiences wishing to disseminate the results of health inequality monitoring in RMNCH or other topics.

### State of inequality: reproductive, maternal, newborn, and child health report

Global level: analysis and reporting

The report and accompanying components are available from: www.who.int/gho/health_equity/report_2015/en/

**Scope**: The report *State of inequality: reproductive, maternal, newborn, and child health* was published in May 2015. The overarching aim of the report is to showcase best practices in reporting the outputs of health inequality monitoring in low- and middle-income countries. The state of inequality encompasses the current and past state of inequality in a country, and thus indicates how a country is performing (level of inequality) and how a country has progressed over time. A secondary aim of the report is to introduce innovative ways for audiences to explore inequality data, including interactive visuals and video clips. These forms of reporting introduce data in a gradual manner that builds from the simple to the complex, and aids interpretation, especially for less technically minded audiences. RMNCH was chosen as the topic for the report due to the availability of disaggregated data that are comparable across countries.

The full report and its electronic components are available both online and in print form with an accompanying CD-ROM. The report was disseminated in all WHO Regions and through other channels in the global public health community.

**Contents**: The *State of inequality: reproductive, maternal, newborn, and child health* report covers 23 health indicators within RMNCH: 17 indicators pertaining to RMNCH interventions, 3 indicators about child malnutrition, and 3 indicators about child mortality. Four dimensions of inequality are applied (economic status, education level, place of residence, and sex), except in the cases of reproductive and maternal health interventions, where it is not relevant to disaggregate by sex. The report contains data from 86 low- and middle-income countries for latest status analyses, and 42 low- and middle-income countries for change over time analyses.

The main body of the report is a collection of feature stories, highlighting selected indicators and dimensions of inequality from subtopics of RMNCH. Each feature story links readers to story point dashboards, which guides them through a series of interactive dashboards and gives them access to all data for that subtopic. Other interactive visuals throughout the report include maps, equity country profiles, and reference tables. In addition, videos are featured throughout the report to illustrate key concepts in monitoring health inequality.

**Intended uses**: The report was primarily developed for those with basic skills in interpreting health-related data. This encompasses a broad audience of technical staff (e.g. in ministries of health), public health professionals, policymakers, researchers, students, and others. The content and principles contained within the report have relevance to those interested in health inequality monitoring, health data communication, novel applications of interactive technologies, and the state of inequality in RMNCH. Specialized knowledge about health inequality and/or previous experience with interactive visualization technologies are not needed to engage with the report.

### Handbook on health inequality monitoring: with a special focus on low- and middle-income countries

Global and national level: collection, analysis, and reporting

The handbook is available from: www.who.int/gho/health_equity/handbook/en/

**Scope**: The *Handbook on health inequality monitoring: with a special focus on low- and middle-income countries* (English version) was published in 2013, and Arabic, Spanish and Portuguese versions of the publication will be released soon. Ultimately, the broad goal of this handbook is to enable the introduction of health inequality monitoring in areas where it is not currently conducted, and to serve as a resource for those working to develop and/or strengthen inequality monitoring systems. The handbook outlines the concepts of health inequality monitoring and elaborates on a stepwise monitoring process using examples from low- and middle-income countries.

The handbook is available online and in print form and has been disseminated in all WHO Regions and within the wider global public health community. In 2014, the *Handbook on health inequality monitoring: with a special focus on low- and middle-income countries* was recognized as ‘highly commended’ in the public health category of the BMA Medical Book Awards.

**Contents**: The *Handbook on health inequality monitoring: with a special focus on low- and middle-income countries* comprises five chapters, and it is roughly organized around the five steps of the monitoring cycle: 1) selecting relevant health indicators and dimensions of inequality; 2) obtaining data; 3) analyzing the data; 4) reporting the results; and 5) implementing changes, where needed, to improve health equity. Emphasis is given to the topics of data sources, measures of health inequality, and reporting practices. The handbook uses data from low- and middle-income countries to explain and apply theoretical concepts throughout, including a comprehensive example of health inequality monitoring from the Philippines. Supplementary boxes within the handbook provide more information to readers in the form of tips, extra information, further readings, and chapter highlights.

A series of eight lectures accompanies the *Handbook on health inequality monitoring: with a special focus on low- and middle-income countries* (available online). The content of the lectures reflects key messages from the full version of the handbook, including a selection of examples and supplementary information.

**Intended uses**: The handbook was developed as a resource for those involved in spearheading, improving, or sustaining national monitoring systems, and was principally intended for use by technical staff of ministries of health. Equally, however, the handbook may be useful to public health professionals, researchers, students, and others, provided that they have basic statistical knowledge and some familiarity with the topic of health monitoring. The handbook was designed to serve as a reference document, and thus does not need to be read from front to back; readers can access the relevant sections of the handbook as per their needs and interests. The contents of the handbook were tailored for national-level monitoring in low- and middle-income countries, but the basic concepts can be applied to any health topic in any country or at any administrative level.

The lecture series may be useful for advanced readers who wish to present the concepts of the handbook to a wider audience. Alternatively, the format of the lecture series may appeal to audiences seeking a concise overview of the concepts.

### Health inequality monitoring eLearning module

Global and national level: collection, analysis, and reporting

The eLearning module is available from: extranet.who.int/elearn/course/category.php?id=15

**Scope**: The *Health inequality monitoring* eLearning module was released in 2015, and it is based on the *Handbook on health inequality monitoring: with a special focus on low- and middle-income countries*. Like the handbook, the eLearning module is an overview of health inequality monitoring, aiming to build theoretical understanding of health inequality monitoring across diverse settings and health topics. The contents of the handbook and eLearning module are similarly organized, and these resources can be easily cross-referenced or explored together.

By presenting this material in the eLearning format, the information is condensed into key messages and discussion points; for further elaboration on points of interest, learners may activate narration and/or notes, or click on elements throughout the course to access more information (i.e. triggering pop-up boxes, video clips, or images).

The *Health inequality monitoring* eLearning module is available online or in hardcopy on CD-ROM. The online module is also available as a ‘no audio/low bandwidth’ version.

**Contents**: This module introduces and explores the general steps of health inequality monitoring, followed by a comprehensive applied example of health inequality monitoring in the Philippines. The module is presented in eight chapters, which are each followed by a number of quiz questions and an application exercise. Quizzes are designed to assess retention of the chapter material, and serve as a tool for the learner to evaluate their progress in the course. The application exercises are open-ended questions that prompt the learner to think about the course material in the context of the health program with which they work and the country, region, or district in which they live. In each chapter, additional information and examples are available to facilitate a more thorough understanding of the material. Additional readings and references are suggested at relevant points throughout the course, and in a separate section at the end of the course.

**Intended uses**: The eLearning module was created for audiences who wish to study health inequality monitoring for practical application within a given setting and context, but also for audiences who wish to gain familiarity with the process of inequality monitoring for other reasons. The concepts outlined within the eLearning module can be applied to measuring inequality in a multitude of health topics and other subject areas.

Presenting the material in an eLearning format provides audiences with an opportunity to engage in self-paced and self-directed learning. Learners can explore relevant sections of the course in any order. All components are optional, and can be completed in whole or in part at the discretion of the learner. The module can be completed in its entirety over the course of several sessions, or in one sitting. The entire module takes approximately 4 h to complete, and is not timed.

### Monitoring health inequality: an essential step for achieving health equity advocacy booklet and video series

Global and national level: collection, analysis, and reporting

The advocacy booklet and video series are available from: www.who.int/gho/health_equity/videos/en/

**Scope**: The advocacy booklet entitled *Monitoring health inequality: An essential step for achieving health equity* and accompanying video series were published in 2014, and then rereleased with minor updates in 2015. These products advocate the importance of health inequality monitoring by communicating simple concepts in a visually appealing and innovative manner. The aim of these products is to draw attention to the need to conduct health inequality monitoring, and encourage resource investments to develop and/or improve these systems. To this end, the advocacy booklet makes use of non-traditional channels – namely QR codes linking to video clips – to promote health information system development.

The advocacy booklet is available online or in hardcopy.

**Contents**: The advocacy booklet outlines four key messages related to health inequality monitoring, which are each accompanied by a figure and short text that support the message. As an extension, a question is posed for each of these examples and answered in a video clip. The booklet also contains general suggestions for improving health information systems.

**Intended uses**: The primary target audience is policymakers, as well as health-related organizations, partnerships, and foundations. These products were designed to have quick appeal to a general audience with no prior exposure to inequality monitoring; accordingly, the content is easily understood and succinct. Viewers must have an appropriate device – such as a smart phone or tablet – to access the videos via the QR codes.

### Capacity building workshops

National level: analysis and reporting

**Scope**: The aim of capacity building workshops is to enable participants to gain skills to do equity analysis and/or to interpret equity data, and to incorporate these practices into health priority setting. Workshops, conducted at the national or regional level, promote national health inequality monitoring, and provide an environment for networking among participants.

To augment the standard capacity building workshop, complementary ‘training of trainers’ (TOT) sessions provide advanced participants with training about the application of workshop material, including advice about how to facilitate subsequent workshops and educational events at the country level.

A series of regional workshops including the TOT component began in April 2014. These workshops promote a systematic and sustainable approach to monitoring health inequality. Three workshops have been hosted by WHO Regions, each reaching several countries: Eastern Mediterranean Region (participating countries were Egypt, Iran, Iraq, Jordan, and Morocco), South-East Asia Region (participating countries were Bangladesh, Bhutan, India, Indonesia, Maldives, Myanmar, Nepal, Sri Lanka, Thailand, and Timor Leste), and Western Pacific Region (participating countries were Cambodia, China, Lao People's Democratic Republic, Mongolia, and Viet Nam).

**Contents**: The capacity building workshops expose participants to the complexities of health inequality monitoring, exploring both theoretical aspects of monitoring and its practical application. Over the course of the workshop, participants become familiar with the process of health inequality monitoring through presentations, demonstrations, hands-on group activities, and plenary discussions. The applied part of the workshops focuses on data analysis and/or interpreting disaggregated data and using them for priority setting. Regional workshops are conducted over the course of 3–4 days.

The TOT component occurs alongside the regional workshop, and TOT participants act as cofacilitators of the regional workshop during group work and discussions. A one-day TOT session precedes the regional workshop. At the end of each day of the regional workshop, the facilitator and TOT participants debrief and discuss the materials from the day, and then review the agenda for the next day. A final TOT session considers how to improve and/or adapt the content of the workshop for future use.

In regional/TOT workshops, participants were provided with disaggregated data from their country on the topic of RMNCH, and gained practical skills in calculating summary measures, preparing inequality reports, and priority setting. Participants used a software application to calculate summary measures of inequality.

**Intended application**: After attending the workshop, participants have the skills to implement or strengthen health inequality monitoring systems at a national level. Participants are adept in interpreting disaggregated data and basic summary measures, and engaging in benchmarking. In particular, participants who completed the TOT education component can contribute to promoting health inequality monitoring within their countries and regions.

Participating countries are encouraged to prepare regular state of inequality reports, using a standardized approach to health inequality monitoring. A post-workshop assessment of inequality, prepared based on the content of the workshop, may serve as an input for a more comprehensive national report. The Philippines, for example, prepared a post-workshop report *Inequality in reproductive, maternal, and child health in the Philippines*, which is available online from: www.who.int/healthinfo/country_monitoring_evaluation/country_activities_Philippines/en/.

***Box 1*. Strengthening equity-oriented health information systems**

**Collection**

The quality and availability of disaggregated data play an important role in determining a country's capacity to monitor inequalities, as they are the inputs for analyses and reporting. Countries should invest resources in developing, strengthening, and/or expanding data collection practices of diverse data sources including household health surveys, facility data, censuses, vital registration systems, and surveillance systems. For example, household health surveys should be introduced in countries where they are not currently conducted, and resources should be devoted to ensuring that they occur on a regular basis; household health surveys can be expanded to cover topics where data are often unavailable, such as non-communicable diseases and injuries.

Whenever possible, data collection should include small-area markers (such as postal codes) or identifiers (such as personal identification numbers) that permit linkages between data sources. Optimally, countries should move toward implementing standardized electronic recordkeeping systems, while ensuring that personal data are protected and used appropriately.

**Analysis**

Countries should focus efforts on developing the technical expertise to conduct health inequality monitoring. Inequality, by nature, is a complex concept that can be calculated using a multitude of different measurements. Conducting health inequality analyses requires not only proficiency in analytical techniques, but also an understanding of their strengths, weaknesses, and appropriate application.

**Reporting**

Reports about health inequality monitoring should always be aligned with the interests, purposes, and technical capacity of the intended audience. Bearing this in mind, reports about health inequality monitoring should aim to communicate clear messages that are supported by underlying data and analyses. Results should be communicated in a manner that is appropriate for the intended audience; data visuals, which may be presented in static or interactive formats, can facilitate the interpretation of large or complex datasets.

## Upcoming work of the WHO

Health equity is a growing priority of the WHO and its Member States, especially as countries work toward achieving the post-2015 SDGs. There is strong impetus for countries to conduct national health inequality monitoring using a standardized, rigorous approach. Thus, the expansion and promotion of the WHO's work at global and national levels, and encompassing all components of the framework (collection, analysis, and reporting) health is both warranted and timely.

As a continuation of its efforts to promote health inequality monitoring, the WHO intends to enable and exemplify health inequality monitoring in a broader scope of health topics, building on its current focus on RMNCH. Indeed, the concepts of health inequality monitoring should be applied to all areas of health where the requisite disaggregated data are available.

The WHO plans to encourage health inequality monitoring at a global level through the following specific initiatives: expand the collection of contents of the *Health Equity Monitor* from RMNCH to include other health topics such as non-communicable diseases, and produce a series of comprehensive reports about the state of inequality in diverse thematic areas (adding to the first report about the state of inequality in RMNCH). At a regional level, the WHO intends to prepare regional specific reports about the state of inequality, focusing on health topics and dimensions of inequality that hold relevance within each WHO Region. At a national level, the WHO intends to continue to conduct training workshops to build capacity for health inequality monitoring, and to provide technical assistance to Member States. In addition, the WHO plans to identify pilot countries to participate in a more intensive process of establishing sustainable, equity-oriented surveillance systems for monitoring health inequality. Pilot countries will be supported in all stages of monitoring, including data collection, analysis, and reporting.

Now is an opportune time for the WHO and its partners to convene to promote health inequality monitoring at global, regional, and national levels. In particular, a global network of research institutes and international experts should collaborate to generate recommendations about health inequality monitoring in the post-2015 period. In addition, such a group could serve as a focal point for providing technical assistance and conducting capacity building activities in countries, contributing to strengthening equity-oriented health information systems.
